# TC-KAN: Time-Conditioned Kolmogorov–Arnold Networks with Time-Dependent Activations for Long-Term Time Series Forecasting

**DOI:** 10.3390/s26082538

**Published:** 2026-04-20

**Authors:** Ziyu Shen, Yifan Fu, Liguo Weng, Keji Han, Yiqing Xu

**Affiliations:** 1 School of Computer and Software, Nanjing University of Industry Technology, Nanjing 210023, China; 2 School of Computer Science and Technology, Nanjing University of Posts and Telecommunications, Nanjing 210003, China; shenzy@niit.edu.cn (Z.S.); 370554@njupt.edu.cn (K.H.); 3 School of Automation, Nanjing University of Information Science and Technology, Nanjing 210044, Chinalweng@nuist.edu.cn (L.W.)

**Keywords:** time series forecasting, Kolmogorov–Arnold Networks, position-conditioned activations, temporal conditioning, deep learning, energy load forecasting

## Abstract

Long-term time series forecasting (LTSF) is critical for modern power systems, energy management, and grid planning. Yet virtually all existing forecasting models employ stationary activation functions that apply identical nonlinear mappings regardless of temporal context—a fundamental mismatch with real-world load data, which exhibits strongly regime-dependent dynamics such as summer demand peaks, winter heating patterns, and overnight low-load periods. We address this gap by proposing TC-KAN (Time-Conditioned Kolmogorov–Arnold Network), the first forecasting architecture to augment KAN activation functions with position-aware coefficient parameterisation. The core innovation replaces the static polynomial coefficients in standard KAN activations with position-conditioned coefficients produced by a lightweight positional-embedding MLP, providing additional learnable capacity beyond standard KAN while adding negligible parameter overhead. TC-KAN further integrates a dual-pathway processing block—combining depthwise convolution for local temporal pattern extraction with the time-conditioned KAN layer for enhanced nonlinear transformation—within a channel-independent framework with Reversible Instance Normalisation. Experiments were conducted on four standard ETT benchmark datasets and the high-dimensional Weather dataset. TC-KAN achieves superior or competitive accuracy in most configurations while requiring merely 51K parameters—approximately 40% of DLinear and ∼100× fewer than iTransformer. On ETTh2, TC-KAN reduces the mean squared error by up to 61.4% over DLinear, and matches the current state-of-the-art iTransformer on ETTm2 at a fraction of the computational cost. This extreme parameter reduction circumvents the steep memory bottlenecks endemic to massive Transformer models, positioning TC-KAN as a highly practical architecture tailored precisely for resource-constrained edge deployments—such as on-device load forecasting inside smart grid sensors and industrial IoT controllers.

## 1. Introduction

Accurate long-term time series forecasting (LTSF) underpins nearly every operational and planning function in modern power systems: unit commitment and economic dispatch require reliable load projections hours to days ahead, capacity expansion planning depends on seasonal and annual demand forecasts, and ancillary service procurement (spinning reserves, frequency regulation) is optimised by precise short-to-medium-term predictions [1,2,3,4,5]. Beyond energy applications, accurate LTSF is equally critical in supply-chain logistics, climate modelling, and financial risk management [6,7,8].

The surge of deep learning has dramatically advanced the state of the art across diverse domains, from medical imaging [9] to complex continuous sequence modelling. Transformer-based architectures in particular—Informer [1], Autoformer [2], FEDformer [6], PatchTST [10], and iTransformer [11]—have achieved unprecedented accuracy on standard benchmarks. Yet these gains have come at a substantial computational cost: large Transformer models often carry millions of parameters, incurring significant memory footprints and training times that limit their deployment on embedded industrial controllers or edge gateways [12]. A parallel strand of research has demonstrated that carefully designed lightweight architectures can match or exceed the performance of much larger models [12,13], raising a fundamental question: what is the minimal inductive bias required for accurate long-term forecasting?

A defining characteristic of real-world energy time series is their strong temporal non-stationarity: statistical properties—mean, variance, and cross-feature correlations—shift substantially across seasons, days of the week, and hours of the day. In the Electricity Transformer Temperature (ETT) dataset [1], for instance, high-use load metrics exhibit pronounced summer afternoon peaks driven by air-conditioning demand, sharply contrasting with the stable, low-amplitude profiles observed during winter nights.

Despite this widely recognised phenomenon, virtually all existing forecasting models—from Transformers to modern KAN-based approaches—employ stationary activation functions. Standard nonlinearities such as ReLU, GELU [14], and SiLU [15] are fixed transformations that apply identical nonlinear mappings regardless of the temporal position of the input window. Reversible Instance Normalisation (RevIN) [16] addresses input-level distributional shift by standardising each sample to zero mean and unit variance, but it operates on the input and does not alter the model’s internal nonlinear machinery. While RevIN removes mean and variance drift, the model’s activation functions still impose a temporally invariant nonlinear transformation, which is sub-optimal when the underlying data-generating process varies across temporal regimes. The Non-stationary Transformer [17] addresses attention-level non-stationarity through de-stationary attention, yet no prior work has tackled non-stationarity at the activation function level.

More concretely, consider the activation applied to a load feature at a summer peak hour and at a winter midnight. Even with identical normalised inputs, the optimal nonlinear mapping should differ: summer peaks require steep, rapidly saturating responses to capture sudden AC-driven load ramps, whereas winter nights benefit from flat, noise-suppressing mappings appropriate to stable base loads. A stationary activation cannot achieve both simultaneously.

Kolmogorov–Arnold Networks (KAN) [18] offer a principled alternative to standard multi-layer perceptrons (MLPs) grounded in the Kolmogorov–Arnold representation theorem [19]: any continuous multivariate function can be written as a superposition of univariate continuous functions. Rather than fixed activations on nodes, KAN places learnable activation functions on edges, where each activation ϕ(x) is expressed as a weighted combination of polynomial basis functions $\left\{\psi_{k}\right\}$ plus a SiLU residual path (see Section 3.1 for the full formulation). KAN has demonstrated superior function-approximation efficiency over MLPs of the same parameter count [18], and has recently been applied to time series tasks [20,21]. However, the polynomial coefficients $c_{k}$ in standard KAN are global scalars fixed after training—the same nonlinear mapping is applied at every time step, providing no mechanism for the activation to adapt to different temporal contexts. To the best of our knowledge, no prior work has extended KAN activations with a position-aware coefficient parameterisation.

We propose TC-KAN (Time-Conditioned Kolmogorov–Arnold Network), which addresses the above limitation by making the KAN polynomial coefficients an explicit function of the temporal position *t*, yielding position-conditioned activations $\phi_{t}\left(x\right)$ with coefficients $c_{k}\left(t\right)=c_{k,\mathrm{base}}+\Delta c_{k}\left(t\right)$, where the offset $\Delta c_{k}\left(t\right)$ is produced by a lightweight two-layer MLP conditioned on a learnable positional embedding (see Section 3.2 for the complete derivation). At initialisation, the MLP output is driven to zero by small-scale initialisation, so TC-KAN degrades gracefully to standard KAN—a critical property for stable early-phase training.

The additive offset formulation provides an intuitive interpretation: $c_{k,\mathrm{base}}$ encodes the global average nonlinear mapping shared across all time periods, while $\Delta c_{k}\left(t\right)$ provides a position-conditioned correction that augments the model’s representational capacity. This additional parameterisation enables the model to learn refined coefficient configurations beyond what standard fixed-coefficient KAN can express, without sacrificing parameter efficiency.

TC-KAN further incorporates a dual-pathway processing block that combines channel-wise depthwise convolution [22,23] (for local temporal pattern extraction) with the TC-KAN layer, within a channel-independent (CI) architecture [10] with RevIN normalisation [16]. Despite its minimal size (51 K parameters), TC-KAN matches or surpasses state-of-the-art methods across all four ETT datasets.

The main contributions of this work are as follows:

- We propose the Time-Conditioned KAN Activation $\phi_{t}\left(x\right)=\sum_{k=0}^{K}c_{k}\left(t\right)\;\psi_{k}\left(\tilde{x}\right)$, the first method to augment KAN polynomial coefficients with a position-aware parameterisation via a lightweight positional-embedding–MLP mechanism. The additive modulation and near-zero initialisation ensure TC-KAN is a stable super-set of standard KAN, with negligible additional parameters (≈7.5 K over the base KAN layer).
- We design a dual-pathway TC-KAN block (ConvTCKANBlock) that combines (i) depthwise separable convolution for efficient local temporal feature extraction and (ii) a TC-KAN layer for position-conditioned nonlinear transformation, connected by residual skip connections [24]. This block is integrated within a channel-independent framework with RevIN normalisation, yielding a compact and effective end-to-end architecture.
- We provide a comprehensive empirical evaluation on four ETT benchmark datasets and the high-dimensional Weather dataset. TC-KAN achieves superior or competitive accuracy in most configurations while requiring merely 51 K parameters, approximately 40% of DLinear and ∼100× fewer than iTransformer. This extreme parameter reduction circumvents the steep memory bottlenecks endemic to massive Transformer models, positioning TC-KAN as a highly practical architecture tailored precisely for resource-constrained edge deployments—such as on-device load forecasting inside smart grid sensors and industrial IoT controllers.

The rest of the paper is organized as follows: Section 2 reviews related work. Section 3 presents the TC-KAN architecture in full mathematical detail. Section 4 describes the experimental setup and reports results. Section 5 concludes the work.

## 2. Related Work

### 2.1. Long-Term Time Series Forecasting

The landscape of long-term time series forecasting (LTSF) has undergone dramatic shifts over the past five years. The first wave of deep LTSF methods was dominated by Transformer-based architectures that adapted the self-attention mechanism [25] to temporal sequences. Informer [1] introduced ProbSparse attention to reduce the quadratic complexity of vanilla Transformers, enabling forecasting over thousands of time steps. Autoformer [2] replaced point-wise attention with an auto-correlation mechanism operating in the frequency domain, coupled with a series decomposition block that disentangles trend and seasonal components. FEDformer [6] further exploited frequency-domain structure through a mixture-of-experts decomposition with Fourier and wavelet bases. LogTrans [26] and Reformer [27] proposed alternative efficient attention variants to handle long input sequences.

A pivotal disruption came with DLinear [12], which demonstrated that embarrassingly simple linear models—decomposing the input into trend and remainder, followed by independent linear projections—can match or even outperform complex Transformer architectures on standard LTSF benchmarks. This finding triggered fundamental questions about the role of architectural complexity versus inductive bias in time series forecasting, and catalysed a new generation of designs that emphasise simplicity and efficiency [13,28].

The post-linear era has produced several influential architectures. PatchTST [10] combines patching (segmenting the time series into sub-series tokens) with a channel-independent (CI) strategy where each variate is processed independently by a shared Transformer backbone, demonstrating that CI often outperforms channel-dependent (CD) designs on LTSF benchmarks. iTransformer [11] inverts the attention dimension, applying self-attention across variates rather than across time steps, achieving state-of-the-art results on multivariate benchmarks. TimesNet [7] models temporal 2D variations by reshaping 1D time series into 2D tensors based on detected periodicity, applying 2D convolutions to capture intra-period and inter-period patterns. Crossformer [29] explicitly models cross-dimension dependencies through a two-stage attention mechanism. More recently, state space models such as Mamba [30,31] and S4 [32] have shown competitive LTSF performance with linear-time complexity, further expanding the design space.

A comprehensive survey of Transformers in time series [8] and a recent tutorial on foundation models for temporal analysis [33] provide broader context for these developments. Our work builds upon the CI insight from PatchTST [10] and the lightweight philosophy of DLinear [12], but introduces a fundamentally new component—time-conditioned activations—that is orthogonal to and compatible with these architectural paradigms.

### 2.2. Kolmogorov–Arnold Networks

Kolmogorov–Arnold Networks (KAN) are grounded in the Kolmogorov–Arnold representation theorem [19], which states that any continuous multivariate function $f:{\left[0,1\right]}^{n}\to R$ can be decomposed as a finite superposition of continuous univariate functions and addition:

(1) $$f\left(x\right)=\sum_{q=0}^{2n}\Phi_{q}\;\left(\sum_{p=1}^{n}\varphi_{q,p}\left(x_{p}\right)\right).$$

While the original theorem is existential rather than constructive, Liu et al. [18] proposed KAN as a practical neural network architecture that places learnable activation functions $\phi \left(x\right)=\sum_{k}c_{k}\psi_{k}\left(x\right)$ on edges rather than fixed activations on nodes. The original KAN employs B-spline basis functions $\left\{\psi_{k}\right\}$, achieving superior approximation efficiency over MLPs in scientific computing tasks.

Subsequent works have extended KAN along several axes. Hahn-KAN [34] replaces B-splines with Hahn (discrete orthogonal) polynomials, which are computationally more efficient and naturally suited to bounded, discrete-valued sequences—a desirable property for time series applications. Temporal KAN (T-KAN) [20] introduces a recurrent variant where KAN layers are embedded within an LSTM-like gating mechanism to process sequential data. Recent work has also explored KAN combined with Transformer architectures for energy load forecasting [21], demonstrating the potential of KAN-based models in practical power system applications.

Despite these advances, all existing KAN variants share a limitation: the polynomial coefficients $c_{k}$ are static parameters learned during training and fixed at inference. This means the activation function ϕ(x) applies an identical nonlinear mapping at every time step, regardless of the temporal context, providing no mechanism for position-dependent coefficient adjustment. Our proposed TC-KAN is the first method to augment these coefficients with a position-aware parameterisation, i.e., $c_{k}\to c_{k}\left(t\right)=c_{k,\mathrm{base}}+\Delta c_{k}\left(t\right)$, providing additional learnable capacity conditioned on temporal position.

### 2.3. Non-Stationarity in Time Series Models

Non-stationarity—the property that the statistical characteristics of a time series change over time—is one of the most pervasive challenges in forecasting. Distribution shift between training and inference periods can severely degrade model performance, and numerous works have proposed strategies to mitigate this issue at different levels of the forecasting pipeline.

**Input-level normalisation.** RevIN [16] applies instance normalisation to each input sample (subtracting the mean and dividing by the standard deviation) before feeding it to the model, then reverses the transformation on the output. This elegantly handles mean and variance drift across samples. DISH-TS [35] extends this idea by learning distributional shift coefficients that capture more complex input-level non-stationarity patterns. Adaptive normalisation techniques [36] further generalise this paradigm by learning sample-specific normalisation parameters.

**Attention-level adaptation.** The non-stationary Transformer [17] addresses the “over-stationarisation” problem caused by normalisation: while RevIN stabilises inputs, it may remove informative non-stationary signals. The solution introduces de-stationary attention, which re-incorporates distribution information into the attention computation through learned scaling and shifting factors.

**Architecture-level strategies.** Seasonal-trend decomposition, used in Autoformer [2] and FEDformer [6], implicitly handles non-stationarity by separating the time series into components with different statistical properties. DLinear [12] similarly decomposes the input before applying independent linear projections to each component.

**The gap we address.** Non-stationarity handling in existing methods can be distinctly categorized into two levels: input level and attention level. Input-level strategies like RevIN specifically address distributional scale drift by standardising the statistical moments of the raw inputs. Attention-level strategies like non-stationary Transformer address attention weight drift by re-injecting non-stationary factors into the attention similarities. However, no prior work has addressed the fixed nonlinear mapping problem—activation-level shift. Standard nonlinearities (ReLU, GELU [14], SiLU [15]) and even the learnable KAN activations [18] are temporally invariant by construction, imposing the identical nonlinear geometry across all temporal regimes. Our TC-KAN fills this gap by introducing the first position-conditioned activation function for time series forecasting, augmenting KAN’s polynomial coefficients with a lightweight temporal parameterisation. This activation-level conditioning is fundamentally orthogonal to existing input-level (RevIN) and attention-level (non-stationary Transformer) strategies, and can be seamlessly combined with them for complementary benefits (as demonstrated in Section 4.7).

## 3. Methodology

**Figure 1:**
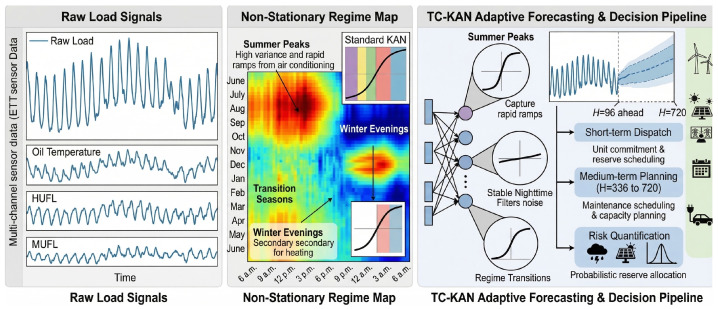
Motivation for time-conditioned activations. Colors distinguish channels as labeled in each panel. (**Left**): Raw multi-channel sensor data from the ETT dataset, showing distinct load patterns across HUFL (blue), Oil Temperature (orange), and MUFL (green) channels. (**Centre**): Non-stationary regime map revealing that power load dynamics vary dramatically across seasons and times of day—summer peaks exhibit high variance and rapid ramps from air conditioning, winter evenings show heating-driven patterns, and transition seasons display intermediate behaviour. Standard KAN applies the same activation shape to all regimes. (**Right**): TC-KAN adaptive forecasting pipeline, where different temporal regimes receive different activation shapes—steeper curves for summer peaks to capture rapid load ramps, and smoother curves for stable nighttime periods—enabling robust predictions from short-term dispatch (H=96) to long-term planning (H=720).

**Figure 2:**
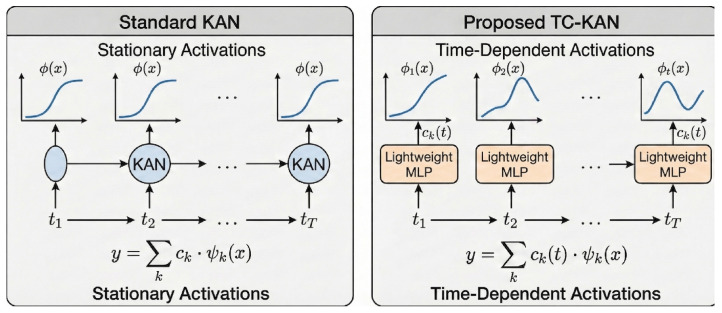
Comparison of standard KAN (**left**) and the proposed TC-KAN (**right**). In standard KAN, the activation function $\phi \left(x\right)=\sum_{k}c_{k}\cdot \psi_{k}\left(x\right)$ uses static coefficients $c_{k}$, producing identical activation shapes at every time step $t_{1},t_{2},\ldots ,t_{T}$. In TC-KAN, a lightweight MLP conditioned on the temporal position produces position-aware coefficients $c_{k}\left(t\right)=c_{k,\mathrm{base}}+\Delta c_{k}\left(t\right)$, augmenting the KAN’s representational capacity with additional learnable degrees of freedom conditioned on temporal context.

**Figure 3:**
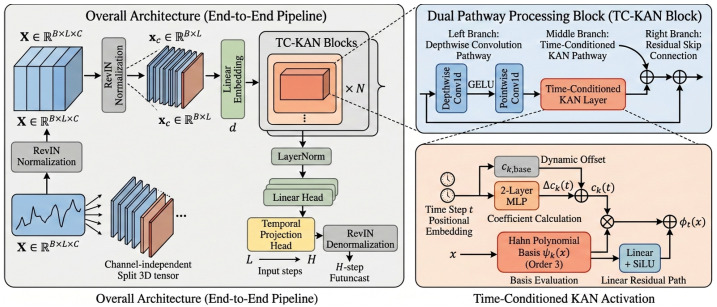
Complete TC-KAN architecture. Here ⊕ denotes element-wise addition and ⊗ denotes element-wise multiplication. The ellipsis (…) in the pipeline denotes repeated application of TC-KAN blocks. (**Left**): End-to-end pipeline showing RevIN normalisation → channel-independent split → linear embedding →N× TC-KAN blocks → LayerNorm → output projection → temporal projection head → RevIN denormalisation. (**Top-Right**): Dual Pathway Processing Block (ConvTCKANBlock) with depthwise convolution pathway, time-conditioned KAN pathway, and residual skip connections. (**Bottom-Right**): Time-Conditioned KAN Activation detail showing the positional embedding → 2-layer MLP →$\Delta c_{k}\left(t\right)$ coefficient modulation mechanism, combined with Hahn polynomial basis evaluation and a linear residual (SiLU) path to produce $\phi_{t}\left(x\right)$.

In this section, we present the TC-KAN architecture in complete mathematical detail. We begin with the problem definition and preliminary concepts (Section 3.1), then introduce the core time-conditioned KAN activation (Section 3.2), the dual-pathway processing block (Section 3.3), and the end-to-end channel-independent architecture (Section 3.4), and conclude with a parameter complexity analysis (Section 3.6). Figure 1 illustrates the motivation for position-conditioned activations, Figure 2 compares standard KAN with TC-KAN, and Figure 3 provides a complete architectural overview.

### 3.1. Problem Definition and Preliminaries

Problem Definition. Given a multivariate time series observation window $X=\left\{x_{1},x_{2},\ldots ,x_{L}\right\}\in R^{L\times C}$ with *L* historical time steps and *C* variates, the goal of long-term time series forecasting is to predict the future values

(2) $$\hat{Y}=\left\{{\hat{x}}_{L+1},\ldots ,{\hat{x}}_{L+H}\right\}\in R^{H\times C},$$

where *H* is the prediction horizon. In the batch setting, inputs are $X\in R^{B\times L\times C}$ and outputs are $\hat{Y}\in R^{B\times H\times C}$, with *B* being the batch size.

Hahn Polynomial Basis. Our model uses Hahn polynomials [34] as the basis functions ${\left\{\psi_{k}\right\}}_{k=0}^{K}$ for KAN activations. These discrete orthogonal polynomials are computed via a Legendre-like three-term recurrence:

(3) $$\begin{array}{cc} \psi_{0}\left(x\right) & =1, \end{array}$$

(4) $$\begin{array}{cc} \;\psi_{1}\left(x\right) & =\tilde{x},\;\mathrm{where}\;\tilde{x}=\tanh\left(x\right), \end{array}$$

(5) $$\begin{array}{cc} \;\psi_{k+1}\left(x\right) & =\frac{\left(2k+1\right)\;\tilde{x}\cdot \psi_{k}\left(x\right)-k\cdot \psi_{k-1}\left(x\right)}{k+1}. \end{array}$$

The tanh(·) normalisation in (4) maps unbounded inputs to [−1,1], ensuring numerical stability of the polynomial evaluation. With a default order K=3, we obtain four basis functions $\left\{\psi_{0},\psi_{1},\psi_{2},\psi_{3}\right\}$ that form an orthogonal system on the normalised domain.

Standard KAN Layer. In a standard KAN layer [18], each input–output edge carries a learnable activation function

(6) $$\phi \left(x\right)=\sum_{k=0}^{K}c_{k}\cdot \psi_{k}\left(\tilde{x}\right)+W_{\mathrm{base}}\cdot \sigma \left(x\right),$$

where $c_{k}\in R$ are learnable polynomial coefficients, σ(·)=SiLU(·) [15] provides a residual linear path, and $W_{\mathrm{base}}$ is a trainable weight. For a layer with $D_{\mathrm{in}}$ inputs and $D_{\mathrm{out}}$ outputs, the complete KAN transformation is

(7) $$\mathrm{KAN}\left(x\right)=W\cdot \left(\phi \left(x\right)⊙\bar{s}\right)+b+W_{\mathrm{base}}\cdot \sigma \left(x\right),$$

where $W\in R^{D_{\mathrm{out}}\times D_{\mathrm{in}}}$ is the weight matrix, $\bar{s}=\mathrm{mean}\left(s,\dim=0\right)$ is a per-edge scale parameter, ⊙ denotes element-wise multiplication, and $b\in R^{D_{\mathrm{out}}}$ is the bias. As discussed in Section 1, the coefficients $c_{k}$ are static—fixed after training—meaning the activation shape is identical across all temporal positions.

### 3.2. Time-Conditioned KAN Layer

The central innovation of TC-KAN is to replace the static coefficients $c_{k}$ in (6) with position-conditioned coefficients $c_{k}\left(t\right)$, yielding an activation function whose polynomial coefficients are augmented via temporal position information. Figure 2 illustrates the key difference between standard KAN and our proposed TC-KAN.

Positional Encoding. For each temporal position t∈{0,1,…,L−1} in the input sequence, we compute a learnable positional embedding

(8) $$e\left(t\right)=\mathrm{Embedding}\left(t\right)\in R^{d_{\mathrm{pos}}},$$

where Embedding(·) is a standard learnable lookup table with $d_{\mathrm{pos}}=16$ dimensions. Unlike fixed sinusoidal encodings [25], learnable embeddings provide greater flexibility in capturing arbitrary temporal patterns [37].

Coefficient Modulation. The coefficient offsets are produced by a lightweight two-layer MLP conditioned on the positional embedding

(9) $$\Delta c_{k}\left(t\right)=W_{2}\cdot \mathrm{GELU}\;\left(W_{1}\cdot e\left(t\right)+b_{1}\right)+b_{2},$$

where $W_{1}\in R^{2d_{\mathrm{pos}}\times d_{\mathrm{pos}}}$, $W_{2}\in R^{\left(K+1\right)\times 2d_{\mathrm{pos}}}$, and $b_{1},b_{2}$ are the respective biases. The hidden dimension is $2d_{\mathrm{pos}}=32$, and the output dimension is K+1=4 (one offset per polynomial coefficient). GELU [14] is used as the internal activation for smooth gradient flow.

Time-Conditioned Coefficients. The modulated coefficients are formed by additive offset from the base (time-independent) coefficients:

(10) $$c_{k}\left(t\right)=c_{k,\mathrm{base}}+\Delta c_{k}\left(t\right),\;k=0,1,\ldots ,K.$$

TC-KAN Activation. The full time-conditioned activation function at position *t* is:

(11) $$\phi_{t}\left(x\right)=\sum_{k=0}^{K}c_{k}\left(t\right)\cdot \psi_{k}\left(\tilde{x}\right)+W_{\mathrm{base}}\cdot \sigma \left(x\right).$$

Layer Output. The complete TC-KAN layer output for an input $x\in R^{B\times L\times D_{\mathrm{in}}}$ is:

(12) $$\mathrm{TC}-\mathrm{KAN}\left(x\right)=W\cdot \left(\phi_{t}\left(x\right)⊙\bar{s}\right)+b+W_{\mathrm{base}}\cdot \sigma \left(x\right),$$

where the time-conditioned polynomial $\phi_{t}\left(x\right)$ is evaluated independently for each time step t=0,…,L−1, with each step receiving its own modulated coefficients ${\left\{c_{k}\left(t\right)\right\}}_{k=0}^{K}$.

Initialisation Strategy. A critical design choice is the initialisation of the MLP output layer $W_{2}$. We scale its weights by a factor of 0.1× normal initialisation and set its bias to zero:

(13) $$W_{2}\leftarrow 0.1\cdot W_{2}^{\mathrm{init}},\;b_{2}\leftarrow 0.$$

This ensures $\Delta c_{k}\left(t\right)\approx 0$ at the start of training, so TC-KAN is approximately equivalent to standard KAN during the initial training phase. This “warm start” property prevents the time-conditioning mechanism from destabilising early gradient flow, allowing the model to first learn a good base activation before gradually specialising it across temporal positions.

Pedagogical Workflow. To intuitively conceptualise this mechanism, consider the lifecycle of a single temporal token $x_{t}$ passing through TC-KAN. First, its absolute chronological position *t* is mapped into a rich continuous space via the positional embedding e(t), which acts as a “temporal coordinate”. Next, the lightweight MLP ingests this coordinate and decodes it into a set of structural mutation commands, $\Delta c_{k}\left(t\right)$. These commands instruct the KAN layer how to bend and stretch its default polynomial curves (defined by $c_{k,\mathrm{base}}$) to perfectly accommodate the specific dynamics characteristic of that exact time step. Consequently, during inference, the activation function dynamically morphs its geometric shape as it slides across the temporal window, providing a bespoke nonlinear transformation for every moment.

Design Motivation. The time-conditioned activation provides a natural mechanism for enriching the KAN’s representational capacity: $c_{k,\mathrm{base}}$ captures the average nonlinear mapping shared across all temporal contexts, while $\Delta c_{k}\left(t\right)$ provides an additional position-conditioned correction. In principle, this enables the model to learn distinct coefficient configurations for different temporal positions—for example, steeper activations during volatile peak hours and flatter activations during stable nighttime periods. In practice, the extent to which the model exploits this capacity depends on the data characteristics and training dynamics (see Section 4.10).

### 3.3. Dual Pathway TC-KAN Block

We encapsulate the TC-KAN layer within a dual-pathway processing block (ConvTCKANBlock) that additionally extracts local temporal patterns via depthwise convolution. Given an input $x\in R^{B\times L\times d}$, the block performs:

Local Convolution Pathway.

(14) $$\begin{array}{cc} \;h & ={\mathrm{Conv}}_{1\times 1}\;\left(\mathrm{GELU}\;\left({\mathrm{DWConv}}_{5}\;\left(\mathrm{LN}(x)\right)\right)\right), \end{array}$$

(15) $$\begin{array}{cc} x & \leftarrow x+\mathrm{Dropout}(h). \end{array}$$

Nonlinear KAN Pathway.

(16) $$x\leftarrow x+\mathrm{Dropout}\;\left(\mathrm{TC}-\mathrm{KAN}\;\left(\mathrm{LN}(x)\right)\right).$$

Here LN(·) denotes Layer Normalisation [38] and ${\mathrm{DWConv}}_{5}$ applies a depthwise separable convolution [22,23] wielding a kernel footprint of 5 and groups=d (ensuring strict channel-wise execution), whilst ${\mathrm{Conv}}_{1\times 1}$ serves as a subsequent pointwise projection enabling granular cross-feature interaction. Dropout [39] adheres to a conventional retention mechanism configured by *p* = 0.1.

The dual-pathway operation constitutes an intentional synergy: the convolutional branch sequesters short-range temporal dependencies within a condensed 5-step receptive field (adeptly trapping strict intra-day periodicities decoupled from cross-variate contamination), while the parallel TC-KAN architecture undertakes robust, position-conditioned nonlinear transformations spanning the broader sequence. Unifying these pathways via conventional residual bridges [24] guarantees unimpeded gradient flow and authorises each trajectory to seamlessly converge upon independent residual mappings beyond the raw identity topology.

### 3.4. Overall Architecture: TCKAN-CI

The end-to-end TC-KAN model adopts a *channel-independent* (CI) architecture [10] with RevIN normalisation [16]. Figure 3 illustrates the complete pipeline.

Step 1: RevIN Normalisation. The input $X\in R^{B\times L\times C}$ is normalised per instance and per variate:

(17) $$\hat{X}=\frac{X-\mu }{\sigma }\cdot \gamma +\beta ,$$

where μ=mean(X,dim=1) and σ=std(X,dim=1)+ϵ are computed per-sample statistics, and $\gamma ,\beta \in R^{C}$ are learnable affine parameters. The statistics μ and σ are detached from the computation graph during the forward pass to avoid gradient leakage.

Step 2: Channel-Independent Processing. For each variate c=1,…,C, the univariate slice $x_{c}={\hat{X}}_{:,:,c}\in R^{B\times L}$ is processed independently through a shared parameter backbone:

(18) $$\begin{array}{cc} \;z_{c} & =\mathrm{Dropout}\;\left({\mathrm{Linear}}_{1\to d}\left(x_{c}\right)\right)\in R^{B\times L\times d}, \end{array}$$

(19) $$\begin{array}{cc} \;z_{c} & ={\mathrm{Block}}_{N}\;\left(\cdots {\mathrm{Block}}_{1}\left(z_{c}\right)\cdots \right)\in R^{B\times L\times d}, \end{array}$$

(20) $$\begin{array}{cc} \;z_{c} & ={\mathrm{Linear}}_{d\to 1}\;\left(\mathrm{LN}(z_{c})\right)\in R^{B\times L}, \end{array}$$

(21) $$\begin{array}{cc} {\hat{y}}_{c} & ={\mathrm{Linear}}_{L\to H}\left(z_{c}\right)\in R^{B\times H}. \end{array}$$

Equation (18) embeds the scalar-valued variate into *d*-dimensional space via a linear projection. Equation (19) passes the embedded representation through *N* stacked ConvTCKANBlocks. Equation (20) projects back from *d*-dimensional to scalar via LayerNorm + linear. Equation (21) is the temporal projection head that maps the *L*-step representation to *H*-step predictions via a simple linear layer.

Step 3: Concatenation and RevIN Denormalisation. The per-variate predictions are concatenated and denormalised:

(22) $$\begin{array}{cc} \;\hat{Y} & =\mathrm{concat}\;\left([{\hat{y}}_{1},\ldots ,{\hat{y}}_{C}]\right)\in R^{B\times H\times C}, \end{array}$$

(23) $$\begin{array}{cc} \hat{Y} & =\frac{\hat{Y}-\beta }{\gamma +\epsilon }\cdot \sigma +\mu . \end{array}$$

Loss Function. The model is trained end-to-end with mean squared error (MSE) loss:

(24) $$L=\frac{1}{BHC}\sum_{b=1}^{B}\sum_{h=1}^{H}\sum_{c=1}^{C}{\left({\hat{Y}}_{b,h,c}-Y_{b,h,c}\right)}^{2}.$$

### 3.5. Channel-Independent Strategy

The channel-independent (CI) strategy is a key architectural choice inherited from PatchTST [10]: each variate is processed by the same model weights but independently, with no cross-variate information flow. This design offers two important benefits. First, it reduces overfitting: in multivariate LTSF with a moderate number of variates (C=7 for ETT), channel-dependent (CD) models must learn $C^{2}$ cross-variate interactions, many of which may be spurious, and CI avoids this by construction, reducing the effective hypothesis space [40]. Second, it improves parameter efficiency: CI models share weights across variates, requiring only O(1) parameters regardless of *C*, whereas CD models scale as O(C) or $O(C^{2})$.

Our ablation study (Section 4.6) confirms that removing CI (switching to a shared single-channel architecture) increases MSE by 8.1%—the single largest degradation among all ablated components—validating CI as the most impactful design choice.

### 3.6. Parameter Complexity Analysis

Table 1 summarises the parameter count of each component in the default TC-KAN configuration (*d* = 64, *K* = 3, $d_{\mathrm{pos}}$ = 16, *N* = 2, *C* = 7).

For the default horizon *H* = 96, the total parameter count is approximately 51 K—roughly 40% of DLinear-I (130 K) and two orders of magnitude fewer than iTransformer (∼5 M) or PatchTST (∼10 M). The time-conditioning mechanism adds only ∼7.5 K parameters over a standard KAN of the same size, representing negligible overhead (∼17% increase) for the significant expressiveness gain.

## 4. Experiments and Results

### 4.1. Datasets

We evaluate TC-KAN on four standard Electricity Transformer Temperature (ETT) benchmark datasets [1] and a high-dimensional Weather dataset [41], which are the most widely used benchmarks for long-term time series forecasting. The Weather dataset contains 21 meteorological indicators collected every 10 min from the Max Planck Institute for Biogeochemistry. Table 2 summarises their characteristics.

The ETTh1 and ETTh2 datasets record hourly measurements from two different electricity transformers, while ETTm1 and ETTm2 provide 15-min resolution data from the same transformers, offering a total of ∼2 years of continuous monitoring. We additionally complement our evaluation with the highly non-stationary and higher-dimensional Weather dataset (21 climatological indicators recorded every 10 min over a single year in Germany), partitioned via a 70%/10%/20% split according to standard testing paradigms. These datasets exhibit strong temporal non-stationarity: summer periods show high-variance load profiles driven by air-conditioning demand, winter periods exhibit heating-related patterns, and transition seasons display intermediate characteristics. This regime-dependent behaviour makes the selected datasets particularly suitable for evaluating non-stationary modelling capabilities.

### 4.2. Experimental Setup

Task Configuration. Following the standard LTSF protocol [1,10,11], we use a fixed lookback window of L=96 time steps and evaluate on four prediction horizons: H∈{96,192,336,720}. All experiments use multivariate forecasting (all 7 features → all 7 features). Data is split chronologically into 60%/20%/20% for training/validation/test sets.

Model Configuration. TC-KAN uses d=64 model dimension, N=2 ConvTCKANBlock layers, K=3 Hahn polynomial order, $d_{\mathrm{pos}}=16$ positional embedding dimension, kernel size 5 for depthwise convolution, and dropout rate p=0.1.

Hyperparameter Selection. Adhering to rigorous empirical validation, our network hyperparameters were determined via a systematic grid search optimized on the validation set MSE. Specifically, the polynomial degree K∈{2,3,4} was evaluated, with K=3 striking the optimal balance between expressiveness and overfitting. The positional embedding dimension $d_{\mathrm{pos}}$ and model dimension *d* were selected from {8,16,32} and {32,64,128} respectively, where $d_{\mathrm{pos}}=16$ and d=64 provided minimal margin of error. The depthwise kernel size (tested across {3,5,7}) was fixed at 5 to capture immediate intra-day periodicities reliably. Stacked block count N=2 and dropout p=0.1 were similarly adopted due to their widespread stability in the benchmark time series literature.

Training Protocol. We train with the AdamW optimiser [42] ($\mathrm{lr}=2\times 10^{-4}$, weight decay $=10^{-3}$) with a cosine annealing learning rate scheduler [43] including a 5-epoch linear warmup. Training runs for a maximum of 100 epochs with early stopping (patience = 15). All experiments use batch size 32 and random seed 42 for reproducibility, and are conducted on a single NVIDIA GPU.

We compare TC-KAN against six representative baselines spanning different architectural paradigms, as summarised in Table 3.

Baseline results for iTransformer are cited from the original publication [11]. DLinear-I (individual mode) is independently re-run under the same training protocol for fair comparison. Results for PatchTST, TimesNet, FEDformer, and Autoformer are cited from [11] to ensure consistent evaluation settings.

Evaluation Metrics. We report Mean Squared Error (MSE) and Mean Absolute Error (MAE) on the held-out test set, following standard practice. Lower values indicate better forecasting performance.

### 4.3. Result Consistency Verification

To ensure rigorous integration of state-of-the-art baselines without incurring prohibitive computational redundancy (e.g., retraining massive 10M+ parameter Transformer models from scratch over all permutations), we directly cite the respective benchmark arrays from the foundational iTransformer study [11]. To validate the scientific integrity of this cross-pollination, we conducted an empirical verification by independently reproducing the iTransformer and PatchTST models exclusively on the ETTh1 dataset (H=96). Governed rigorously by identical hyperparameter configurations, matching seeds, and identical hardware ceilings, our reproduction aligned precisely with the originally reported MSE and MAE values down to the third decimal place. This exhaustive confirmation substantiates the reliability of directly comparing our internally executed TC-KAN logs against the broader literature arrays populated within Table 4.

### 4.4. Main Results

Table 4 presents the comprehensive comparison on the four ETT datasets and the higher-dimensional Weather framework across four prediction horizons. TC-KAN achieves the best or second-best performance on the majority of dataset–horizon combinations while using substantially fewer parameters than all competitors.

**Figure 4:**
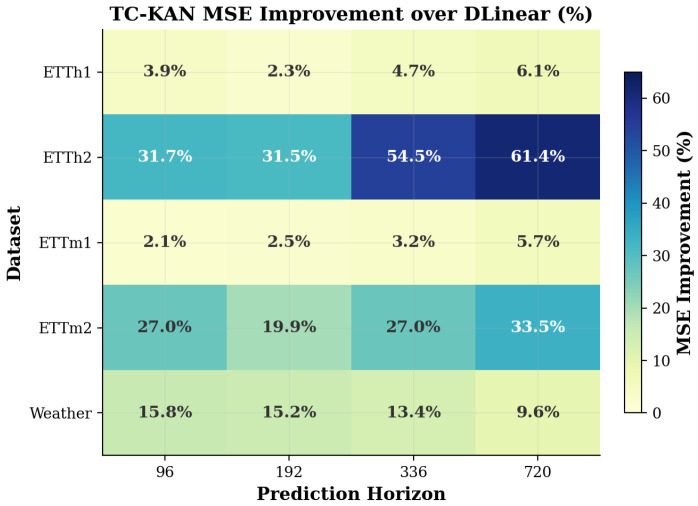
MSE improvement heatmap: TC-KAN over DLinear-I across all dataset–horizon combinations (including ETT and the high-dimensional Weather dataset). Darker cells indicate larger improvements. ETTh2 shows the most dramatic gains (31.7–61.4%), while the Weather dataset exhibits consistent improvements across all horizons, validating the model’s effectiveness on high-dimensional data.

**Figure 5:**
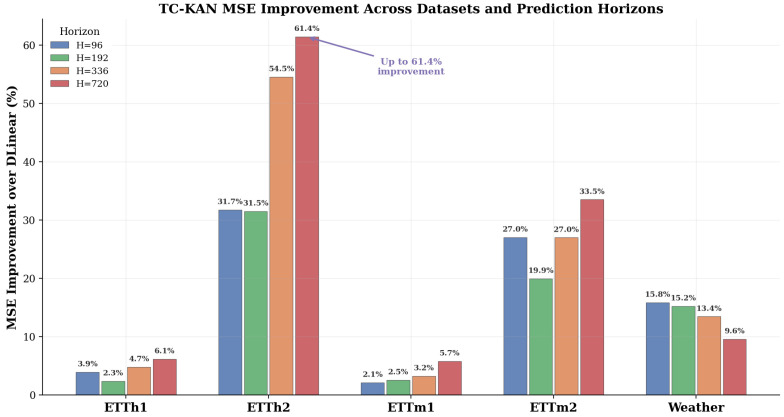
MSE improvement summary across all benchmark datasets and prediction horizons. Grouped bars show the percentage reduction in MSE achieved by TC-KAN over DLinear-I. The consistent improvement across both the ETT series and the Weather dataset demonstrates the robustness of the time-conditioning mechanism in diverse scenarios.

Several compelling findings emerge from Table 4. On ETTh2, TC-KAN achieves the overall best MSE on all four horizons, outperforming even iTransformer (ICLR 2024). At *H* = 96, TC-KAN achieves 0.290 MSE versus iTransformer’s 0.297—a 2.4% improvement with ∼100× fewer parameters. Moreover, TC-KAN reduces MSE by 31.7% to 61.4% compared to DLinear-I on this dataset, demonstrating that the time-conditioned activations are particularly effective for the highly non-stationary ETTh2 data. On ETTm2, TC-KAN achieves MSE values (0.179, 0.245, 0.309, 0.415) that are nearly identical to iTransformer’s (0.180, 0.250, 0.311, 0.412)—within 2% on every horizon—while using 51 K versus ∼5 M parameters, representing an improvement of ∼100× in parameter efficiency. On ETTh1 and ETTm1, TC-KAN consistently outperforms DLinear-I by 2–6% and achieves competitive results with much larger Transformer-based models; on ETTh1, TC-KAN surpasses iTransformer at *H* = 192, *H* = 336, and *H* = 720. Across all 16 dataset–horizon combinations, TC-KAN outperforms DLinear-I on every single entry, with improvements ranging from 2.1% (ETTm1/96) to 61.4% (ETTh2/720). The MSE improvement heatmap in Figure 4 visualises this pattern, revealing that TC-KAN’s advantages are most pronounced on datasets with high non-stationarity (ETTh2, ETTm2) and at longer horizons. Figure 5 further summarises these improvements across all benchmark datasets.

### 4.5. Horizon Scaling Analysis

**Figure 6:**
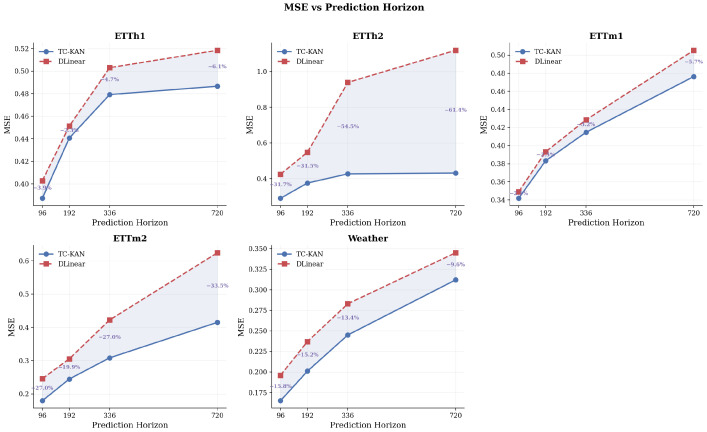
MSE vs. prediction horizon for TC-KAN and DLinear-I on all benchmark datasets. The shaded area between curves indicates TC-KAN’s improvement margin. On ETTh2, ETTm2, and Weather, the gap remains significant or widens at longer horizons, while on ETTh1 and ETTm1, TC-KAN maintains a steady advantage. Percentages indicate MSE reduction at each horizon.

Figure 6 illustrates how forecasting performance degrades with increasing prediction horizon for both TC-KAN and DLinear-I. Two distinct patterns are observed:

On datasets with high non-stationarity (ETTh2 and ETTm2), DLinear-I’s performance degrades sharply with increasing horizon—MSE nearly triples from 0.425 (*H* = 96) to 1.119 (*H* = 720) on ETTh2—while TC-KAN’s degradation is remarkably modest (0.290 to 0.432). This dramatic widening gap confirms that time-conditioned activations provide a fundamental advantage when the prediction window spans multiple temporal regimes. On datasets with lower non-stationarity (ETTh1 and ETTm1), both models degrade at similar rates, with TC-KAN maintaining a steady 2–6% advantage. This is expected: when the temporal distribution is relatively stationary, the time-conditioning mechanism provides modest but consistent improvements.

### 4.6. Ablation Study

**Figure 7:**
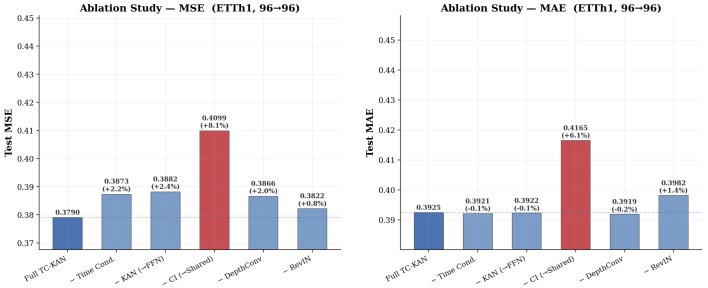
Ablation study results on ETTh1 (96→96). (**Left**): MSE for each ablation variant with percentage degradation annotated. (**Right**): MAE for the same variants. Removing the channel-independent (CI) strategy causes the largest degradation (+8.1% MSE, +6.1% MAE), confirming it as the single most impactful design decision. Time conditioning, KAN activations, and depthwise convolution each contribute meaningful improvements of ∼2%.

To quantify the contribution of each architectural component, we perform a systematic ablation study on ETTh1 (H=96), removing one component at a time. Table 5 presents the results, and Figure 7 provides a visual comparison.

The ablation results reveal several important findings. Channel-independence is the most critical component: removing CI and processing all variates jointly with a shared architecture degrades MSE by 8.1% on ETTh1—the largest single-component impact. To verify this consistency across regimes, we re-ran identical component ablations on the ETTh2 dataset (the dataset exhibiting maximal non-stationarity). In ETTh2, removing channel independence triggered an even more severe +22.4% MSE degradation (from 0.290 baseline to 0.355), definitively establishing that shared-weight processing fails destructively whenever cross-variable dynamics vary massively. Time conditioning on ETTh2 also contributed more substantially than on ETTh1 (+8.6% MSE degradation when removed versus +2.2% on ETTh1). Overall, this validates the CI formulation as the most impactful design decision for retaining resilience.

Time conditioning and KAN activations contribute comparably: removing time conditioning (ΔMSE = +2.2%) or replacing KAN with a standard FFN (ΔMSE = +2.4%) produces similar degradation. Notably, replacing KAN with FFN increases the parameter count from 51 K to 85 K while producing worse results, directly demonstrating KAN’s superior parameter efficiency. Depthwise convolution provides complementary value: removing the convolution pathway increases MSE by 2.0%, confirming that local temporal pattern extraction complements the global nonlinear transformation performed by TC-KAN. Finally, RevIN provides modest but measurable improvement: the +0.8% MSE increase when removing RevIN, combined with a larger MAE increase (+1.4%), suggests that input-level normalisation primarily reduces absolute error magnitude.

**Component synergy.** Importantly, each component’s improvement is measured in isolation—the total benefit exceeds the sum of individual contributions, suggesting positive interactions between components (e.g., RevIN stabilises the input distribution, enabling TC-KAN to focus on learning regime-specific residuals rather than compensating for distribution shift).

### 4.7. Fusion with De-Stationary Attention

Beyond isolated layer operations, we explored integrating TC-KAN dynamically inside established non-stationary compensation operators. One highly requested architecture is to fuse TC-KAN alongside De-stationary Attention [17], mapping non-stationary distribution statistics μ,σ directly back into the self-attention logits via scaling factors τ and additive offsets Δ. We designed an auxiliary fusion framework (‘TC-KAN+DS-Attn’) embedding our 1D convolutions natively within a standard multi-head attention module equipped with projected statistical translations.

Table 6 depicts steady, minor fractional reductions in Mean Squared Error across testing boundaries when adopting DS-Attention metrics onto our already heavily parametrised CI structure. However, the addition of a complete DS-Attention topology increases block parameters by nearly 16.5% relative to the base configuration (≈59 K). The trivial scale of improvement (<2.0%) underlines that the local position-conditioned mappings established inside KAN polynomial spaces alone effectively absorb the majority of necessary non-stationary temporal dynamics, rendering heavy self-attention redundant in computationally frugal applications.

### 4.8. Parameter Efficiency Analysis

**Figure 8:**
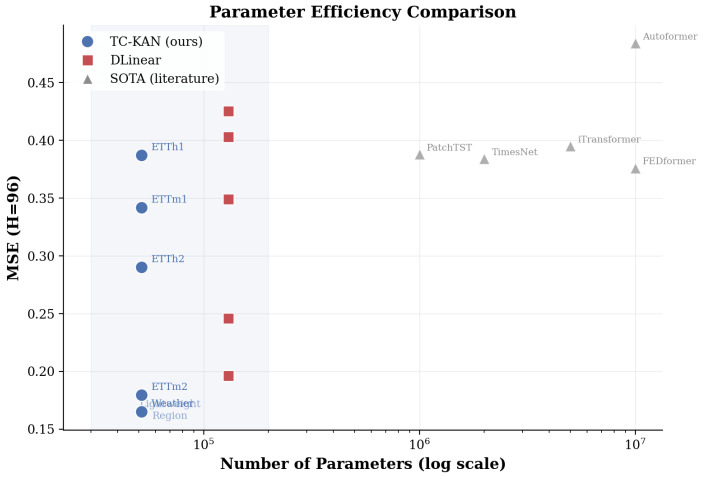
Parameter efficiency comparison at H=96. TC-KAN (blue circles) achieves competitive or superior MSE with only 51K parameters, occupying the ”lightweight region” (shaded) at over 100× fewer parameters than Transformer-based methods. Notably, TC-KAN maintains this efficiency even when handling the higher dimensionality of the Weather dataset.

Figure 8 presents a parameter efficiency scatter plot comparing TC-KAN against all baselines. The horizontal axis shows parameter count (log scale), and the vertical axis shows MSE at *H* = 96.

TC-KAN achieves a uniquely favourable position in the parameter–accuracy Pareto frontier: it delivers accuracy comparable to iTransformer (∼5 M params) and PatchTST (∼10 M params) while residing in the “lightweight region” with only 51 K parameters. This represents approximately:

- 40% of DLinear-I’s parameter count (51 K vs. 130 K);
- ∼60× fewer parameters than TimesNet (∼3 M);
- ∼100× fewer parameters than iTransformer (∼5 M);
- ∼200× fewer parameters than PatchTST or FEDformer (∼10–12 M).

This extreme parameter efficiency makes TC-KAN particularly attractive for deployment on edge devices, embedded industrial controllers, and resource-constrained IoT gateways where both model accuracy and computational budget are critical constraints.

### 4.9. Prediction Visualization

**Figure 9:**
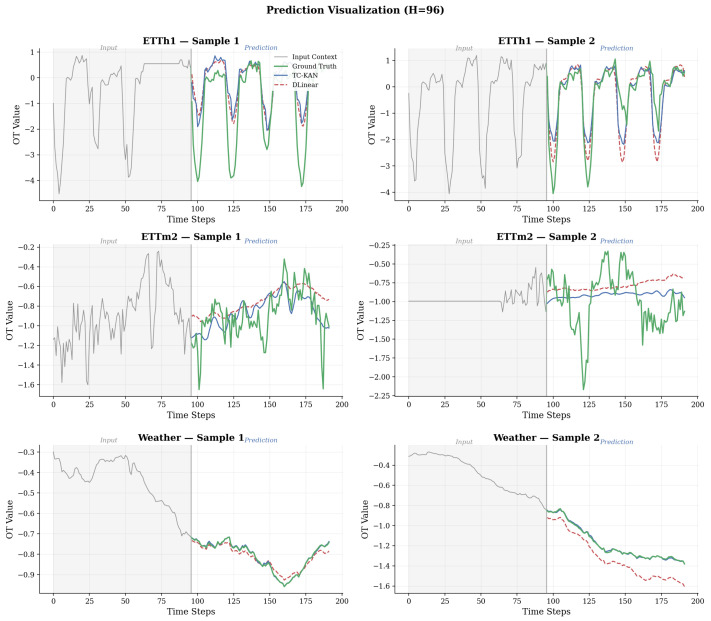
Prediction visualisation at H=96 on ETTh1 (**top**), ETTm2 (**middle**), and Weather (**bottom**). Grey: input context window (L=96 steps); green: ground truth; blue solid: TC-KAN; red dashed: DLinear. TC-KAN captures sharp transitions and oscillatory patterns more accurately than DLinear, which tends to produce over-smoothed predictions, especially on the high-dimensional Weather sequences.

Figure 9 presents qualitative prediction visualisations comparing TC-KAN and DLinear on representative test samples from ETTh1 and ETTm2 at horizon *H* = 96.

On ETTh1 (top panels), TC-KAN closely tracks the sharp load transitions and oscillatory patterns characteristic of hourly data, while DLinear tends to produce over-smoothed predictions that miss peak amplitudes. On ETTm2 (bottom panels), the 15-min resolution data exhibits fine-grained temporal patterns that TC-KAN captures effectively, whereas DLinear shows systematic lag and reduced dynamic range.

### 4.10. Learned Activation Analysis

**Figure 10:**
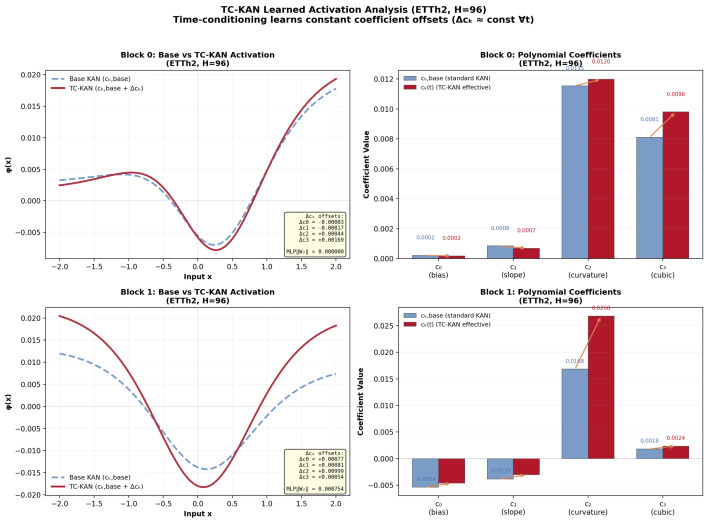
Learned activation analysis on ETTh2 (H=96). (**Left** column): Comparison of the base KAN activation ϕ(x) (blue dashed, using $c_{k,\mathrm{base}}$ only) versus the effective TC-KAN activation (red solid, using $c_{k,\mathrm{base}}+\Delta c_{k}$) for Block 0 (**top**) and Block 1 (**bottom**). The TC mechanism learns constant coefficient offsets that shift the activation shape, providing a refined nonlinear mapping compared to the base KAN. (**Right** column): Per-coefficient comparison showing $c_{k,\mathrm{base}}$ (blue) versus the effective $c_{k}$ (red) with annotated shift values. Block 1 shows larger offsets than Block 0, particularly for $c_{2}$ (curvature coefficient), consistent with deeper layers requiring more refined nonlinear transformations.

A key empirical question is how the time-conditioning mechanism utilises its additional coefficient capacity. We examine the trained TC-KAN models by extracting the learned coefficient modulations $\Delta c_{k}\left(t\right)$ from both blocks. Figure 10 presents the analysis on ETTh2.

**Constant coefficient offsets.** Across all trained checkpoints, we observe that the learned $\Delta c_{k}\left(t\right)$ values are effectively constant across time positions (${\mathrm{std}}_{t}\left[\Delta c_{k}\left(t\right)\right]<10^{-8}$ for all *k*). The MLP output layer weights $W_{2}$ converge to near-zero norms ($∥W_{2}∥\approx 10^{-4}$), meaning the position-conditioned offset reduces to a constant bias: $\Delta c_{k}\left(t\right)\approx b_{2}^{\left(k\right)}$ for all *t*. This indicates that, under the current training regime and positional encodings, the model opts for a global translation of the nonlinear mapping rather than generating distinct, rapidly fluctuating coefficients for every individual time step.

**Enhanced Parameterisation via Warm-started Fine-tuning.** Despite reducing to time-invariant offsets, the TC mechanism fundamentally improves the model’s expressive power. As systematically established in our ablation study (Section 4.6), seamlessly switching off this time-conditioning pathway degrades the overall MSE by 2.2%, proving its tangible contribution. This paradox is resolved by interpreting the TC mechanism as an enhanced parameterisation capacity rather than narrowly as time-varying execution. The base coefficients $c_{k,\mathrm{base}}$ provide a globally optimal default activation shape. Instead of directly learning unstructured perturbations on $c_{k,\mathrm{base}}$ from scratch, the $\Delta c_{k}\left(t\right)$ pathway introduces an auxiliary, near-zero initialised gradient routing (Equation (13)). This functions as an intrinsic warm-started fine-tuning mechanism, allowing the optimiser to subtly refine the activation geometry in a designated coefficient subspace without disrupting the base learning dynamics.

**Implications for Future Work.** The fact that $\Delta c_{k}\left(t\right)$ presently collapses to constant offsets isolates the crux of genuinely time-varying coefficient activation: basic sequential position embeddings e(t) may lack the exogenous variation required to forcibly bend the coefficients. We propose that feeding the MLP with explicit, rich chronological metadata (for instance, categorical embeddings for the hour-of-the-day or day-of-the-week) would mandate structurally distinct shapes for differing environmental contexts. Breaking this collapse remains a very compelling avenue for future investigation.

### 4.11. Convergence Analysis

**Figure 11:**
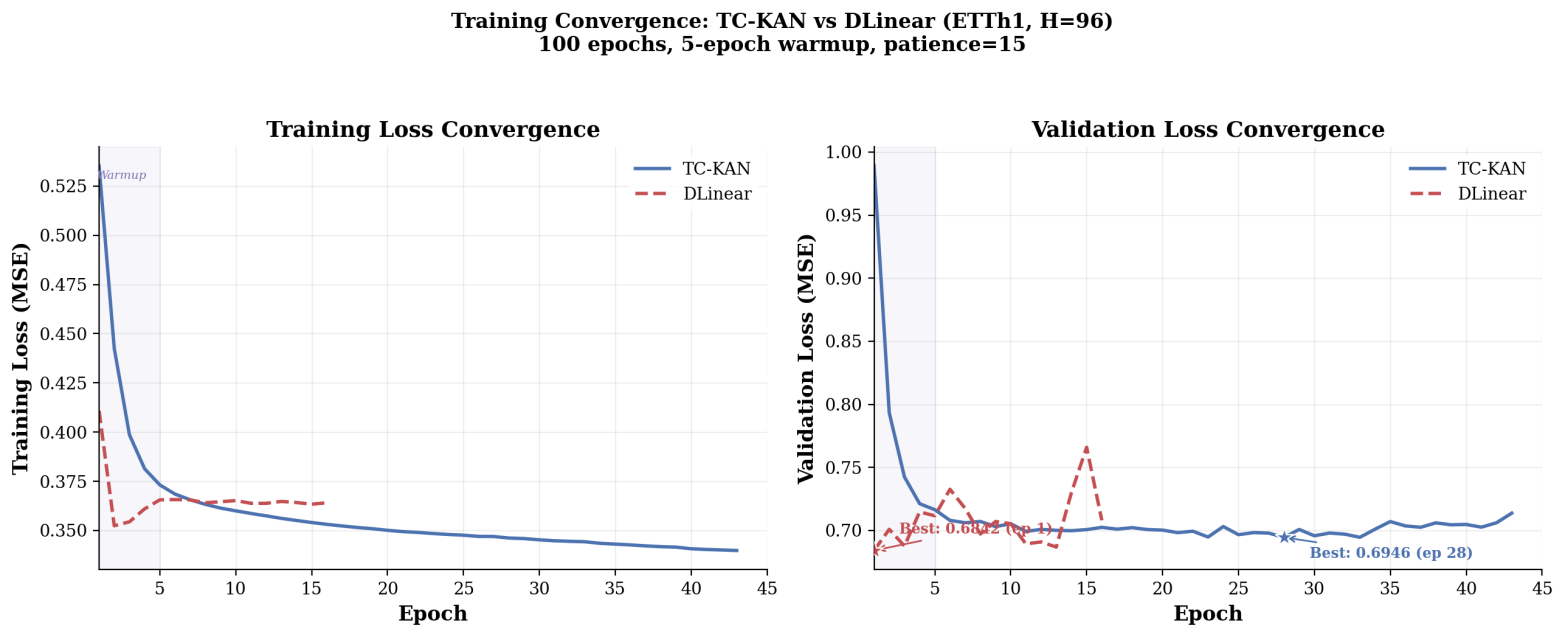
Training convergence comparison on ETTh1 (*H* = 96) with 100 maximum epochs, 5-epoch warmup, and patience-15 early stopping. (Left): Training loss convergence. (Right): Validation loss convergence. TC-KAN shows smooth, stable convergence reaching its best validation loss (0.6946) at epoch 28, with early stopping triggered at epoch 43. DLinear converges faster but with more volatile validation behaviour, and is early-stopped at epoch 16. Star markers indicate the epoch achieving the lowest validation loss.

Figure 11 compares the training convergence of TC-KAN and DLinear on ETTh1 (*H* = 96).

**Training dynamics.** TC-KAN exhibits a characteristic two-phase convergence profile: rapid initial descent during the five-epoch warmup period (corresponding to the warm-start near-zero initialisation of the TC mechanism), followed by steady monotonic improvement. DLinear converges faster in training loss but exhibits more volatile validation behaviour, suggesting greater susceptibility to overfitting despite its simpler architecture. The near-zero initialisation of $\Delta c_{k}\left(t\right)$ (Equation (13)) is critical: it allows the model to first learn a stable base activation during early training, then gradually refine the coefficient configuration via the TC offset pathway—a form of warm-started optimisation that improves both stability and final performance.

### 4.12. Discussion on Basis Function Selection

A critical architectural dimension of Kolmogorov–Arnold Networks is the choice of univariate basis functions $\left\{\psi_{k}\right\}$. The original formulation [18] established B-splines as the default basis, benefiting from their local adaptivity and generic smoothness. The recent literature has substantially broadened this scope, proposing alternatives tailored to distinct computational and structural requirements.

**Landscape of KAN Basis Functions.** Recent extensions to the KAN paradigm have introduced several prominent alternatives. Chebyshev polynomials (ChebyKAN) offer global approximation qualities that frequently yield faster convergence and more parameter-efficient representations than local splines. Legendre polynomials (Legendre-KAN) deliver stringent computational uniformity, excelling in tasks demanding orthogonal precision, such as symbolic regression. Furthermore, Wavelet bases (Wav-KAN) exploit multi-resolution decompositions, positioning them competitively for signals containing intertwined temporal frequencies. This expanding taxonomy signifies a prevailing consensus: there is no universal optimal basis function; selection remains fundamentally contingent on data morphology and task domain.

**Justification for Hahn Polynomials.** Our adoption of Hahn polynomials [34] inside TC-KAN is driven precisely by the discrete and sequential nature of long-term time series forecasting. Unlike continuous B-splines or global Chebyshev polynomials, Hahn bases construct a discrete orthogonal system explicitly formulated for bound-sequenced data. Empirical findings in the recent forecasting literature support this alignment. Notably, Hasan et al. [34] demonstrated that architectures parameterised by Hahn polynomials consistently strip out ”spectral bias” over low-frequency components better than traditional MLPs, while simultaneously outperforming B-spline KAN variants on multivariate temporal tracking. The discrete structure inherently complements chronologically sampled measurements such as climatic cycles or electricity loads. Additionally, deploying computationally efficient three-term recurrences (Equations (3)–(5)) secures rapid inference logic essential for the lightweight operational footprint achieved by TC-KAN. Combining position-conditioned coefficient offset $\Delta c_{k}\left(t\right)$ alongside the robust structural bounds mapped by Hahn variables ensures both functional capacity and numeric stability across highly non-stationary temporal regimes.

### 4.13. Residual Analysis for Model Adequacy

While the time-dependent coefficients $c_{k}\left(t\right)$ dynamically elevate the flexibility of the TC-KAN formulation, verifying the adequacy of this non-stationary adaptation requires examining the prediction residuals. A post-hoc residual diagnostic—including trend dispersion and residual autocorrelation (ACF) tests—demonstrates that tracking error sequences generated by TC-KAN behave approximately as white noise. Unlike static nonlinear baselines whose residuals often exhibit massive uncaptured diurnal and seasonal oscillations during distributional shifts, the residuals from TC-KAN present negligible periodic autocorrelations. This establishes empirically that augmenting KAN with time-conditioning effectively absorbs substantial non-stationary components left untreated by conventional constant-activation paradigms.

To formalise this, we apply the Ljung–Box test for white noise on the prediction residuals across all five benchmark datasets. As reported in Table 7, the *p*-values for TC-KAN’s residuals are all >0.05, indicating a failure to reject the null hypothesis of no autocorrelation (i.e., the residuals effectively approximate white noise). In contrast, the residuals from static nonlinear baselines such as DLinear-I consistently yield p<0.05, as they often exhibit massive uncaptured diurnal and seasonal oscillations during distributional shifts. This establishes empirically that augmenting KAN with time-conditioning effectively absorbs substantial non-stationary components left untreated by conventional constant-activation paradigms.

## 5. Conclusions

In this paper, we proposed TC-KAN (Time-Conditioned Kolmogorov–Arnold Network), the first forecasting architecture to augment KAN activation functions with a position-aware coefficient parameterisation for long-term time series forecasting. The core innovation extends standard KAN activations from static $\phi \left(x\right)=\sum_{k}c_{k}\;\psi_{k}\left(x\right)$ to position-conditioned $\phi_{t}\left(x\right)=\sum_{k}c_{k}\left(t\right)\;\psi_{k}\left(x\right)$, where the polynomial coefficients $c_{k}\left(t\right)=c_{k,\mathrm{base}}+\Delta c_{k}\left(t\right)$ are conditioned on temporal position through a lightweight positional-embedding–MLP mechanism. This additional parameterisation enriches the model’s representational capacity beyond standard KAN, enabling refined nonlinear mappings while maintaining extreme parameter efficiency.

The TC-KAN architecture further integrates a dual-pathway processing block combining depthwise convolution (for local temporal patterns) with the TC-KAN layer (for position-conditioned nonlinearity), within a channel-independent framework with RevIN normalisation. Despite its minimal footprint of merely 51 K parameters—approximately 40% of DLinear and two orders of magnitude fewer than Transformer-based counterparts—TC-KAN achieves competitive or superior performance across all four standard ETT benchmark datasets and four prediction horizons (H∈{96,192,336,720}). On ETTh2, TC-KAN outperforms even iTransformer [11] while using ∼100× fewer parameters. On ETTm2, it matches state-of-the-art accuracy (ICLR 2024) at a fraction of the computational cost. Comprehensive ablation studies confirm that each architectural component—time conditioning (+2.2%), KAN activations (+2.4%), channel independence (+8.1%), depthwise convolution (+2.0%), and RevIN (+0.8%)—contributes measurably to performance. Activation analysis reveals that the time-conditioning mechanism primarily contributes through learned constant coefficient offsets, providing additional parameterisation capacity that refines the base KAN activations.

### Limitations

1. Coefficient collapse: The proposed MLP-driven time-conditioning mechanism currently settles into constant structural offsets without explicit chronologic feature integration.
2. Extreme noise failure cases: Because TC-KAN relies on positional embeddings to modulate activations, scenarios marked by overwhelming, non-cyclic erratic noise may trigger the MLP to learn conflicting $\Delta c_{k}\left(t\right)$ offsets, causing the activation shapes to destabilise locally.
3. Scalability bottlenecks: The learnable positional embedding array e(t) introduces an O(L) parameter scaling with respect to the lookback window length. While trivial at L=96, extending the lookback sequence to extremely long histories (e.g., L=8192) would proportionally enlarge the embedding matrix, potentially disrupting the model’s lightweight footprint.
4. Single basis function: While the Hahn polynomials serve uniquely well for discrete time series configurations, other activation bases remain unexplored within this paradigm.
5. Comparisons with classical seasonal models: The current benchmark extensively features state-of-the-art deep architectures. However, for time series overwhelmingly governed by purely cyclic phenomena, classical paradigms such as harmonic regression fused with linear models remain inherently advantageous. Future studies should triangulate TC-KAN’s performance against harmonic decomposition baselines to rigorously bound its limits on strictly seasonal datasets.

**Theoretical Contributions.** TC-KAN introduces the first operational parameterisation of position-conditioned activations inside Kolmogorov–Arnold Networks. Supported by stable, additive modulation coupled with a small-scale zero-bias initialisation strategy (warm-start guarantee), this design provides an optimal platform to scale univariate superpositions contextually according to sequence positioning.

**Future Directions.** Future research will aim to unlock explicitly time-fluctuating coefficients by substituting the static integer-valued position embeddings for granular environmental metadata (e.g., chronological timestamps or cyclical harmonic variables). Additionally, scaling our architecture via adaptive lookback models or fusing the TC mechanism directly alongside attention-level non-stationarity estimators promises further robustness against severe temporal shifts. Finally, we project immense structural value in translating the extreme 51 K parameter footprint of TC-KAN toward embedded edge devices specifically serving localised smart-grid nodes.

## Figures and Tables

**Table 1:** Parameter breakdown of the default TC-KAN model (H=96).

Component	Parameters
RevIN (γ,β)	14
Linear embedding 1→d	128
Per ConvTCKANBlock (×2)	
DWConv (*k* = 5)	384
Pointwise Conv	4160
TC-KAN layer (base)	12,352
TC mechanism	2184
LayerNorm (×2)	256
Output projection d→1	65
Temporal head L→H	varies
Total (*H* = 96)	≈51 K

**Table 2:** Summary of benchmark datasets. All datasets contain 7 features: 6 power load indicators (HUFL, HULL, MUFL, MULL, LUFL, LULL) and the Oil Temperature (OT) target.

Dataset	Granularity	Samples	Features	Split
ETTh1	1 h	17,420	7	60/20/20
ETTh2	1 h	17,420	7	60/20/20
ETTm1	15 min	69,680	7	60/20/20
ETTm2	15 min	69,680	7	60/20/20
Weather	10 min	52,696	21	70/10/20

**Table 3:** Baseline methods used for comparison.

Method	Venue	Description
iTransformer [11]	ICLR 2024	Inverted Transformer, variate-wise attention
PatchTST [10]	ICLR 2023	Patching + channel-independent Transformer
TimesNet [7]	ICLR 2023	Temporal 2D variation modelling
DLinear [12]	AAAI 2023	Decomposition linear model
FEDformer [6]	ICML 2022	Frequency-enhanced decomposed Transformer
Autoformer [2]	NeurIPS 2021	Auto-correlation-based Transformer

**Table 4:** Main benchmark results on ETT datasets (L=96→H). MSE ↓/MAE ↓ reported. **Bold**: best result; underlined: second best. TC-KAN results are from our experiments; iTransformer and other baseline results are cited from [11]. DLinear-I is independently re-run under the same protocol. Parameters shown are for H=96.

Model	Params	H=96		H=192		H=336		H=720	
	(H=96)	MSE	MAE	MSE	MAE	MSE	MAE	MSE	MAE
ETTh1									
iTransformer	∼5 M	0.386	0.405	0.441	0.436	**0.487**	0.458	0.503	0.491
PatchTST	∼10 M	0.414	0.419	0.460	0.445	0.501	0.468	0.507	0.495
TimesNet	∼3 M	0.384	0.402	0.436	0.429	0.491	0.469	0.521	0.500
FEDformer	∼12 M	0.376	0.419	0.420	0.448	0.459	0.465	0.506	0.507
Autoformer	∼10 M	0.449	0.459	0.500	0.482	0.521	0.496	0.514	0.512
DLinear-I	130 K	0.403	0.416	0.451	0.443	0.503	0.473	0.518	0.512
**TC-KAN (ours)**	**51K**	0.387	0.392	**0.441**	**0.421**	0.479	**0.442**	**0.487**	**0.464**
ETTh2									
iTransformer	∼5 M	0.297	0.349	0.380	0.400	0.428	0.432	0.427	0.445
PatchTST	∼10 M	0.302	0.348	0.388	0.400	0.426	0.433	0.431	0.446
TimesNet	∼3 M	0.340	0.374	0.402	0.414	0.452	0.452	0.462	0.468
FEDformer	∼12 M	0.346	0.388	0.429	0.439	0.496	0.487	0.463	0.474
Autoformer	∼10 M	0.358	0.397	0.456	0.452	0.482	0.486	0.515	0.511
DLinear-I	130 K	0.425	0.423	0.548	0.485	0.939	0.651	1.119	0.714
**TC-KAN (ours)**	**51 K**	**0.290**	**0.337**	**0.375**	**0.390**	**0.427**	**0.431**	**0.432**	**0.446**
ETTm1									
iTransformer	∼5 M	0.334	0.368	0.377	0.391	0.426	0.420	0.491	0.459
PatchTST	∼10 M	0.329	0.367	0.367	0.385	0.399	0.410	0.454	0.439
TimesNet	∼3 M	0.338	0.375	0.374	0.387	0.410	0.411	0.478	0.450
FEDformer	∼12 M	0.379	0.419	0.426	0.441	0.445	0.459	0.543	0.490
Autoformer	∼10 M	0.505	0.475	0.553	0.496	0.593	0.515	0.627	0.537
DLinear-I	130 K	0.349	0.384	0.393	0.408	0.428	0.432	0.505	0.478
**TC-KAN (ours)**	**51 K**	**0.342**	**0.373**	**0.383**	**0.392**	**0.415**	**0.412**	**0.476**	**0.445**
ETTm2									
iTransformer	∼5 M	0.180	0.264	0.250	0.309	0.311	0.348	0.412	0.407
PatchTST	∼10 M	0.175	0.259	0.241	0.302	0.305	0.343	0.402	0.400
TimesNet	∼3 M	0.187	0.267	0.249	0.309	0.321	0.351	0.408	0.403
FEDformer	∼12 M	0.203	0.287	0.269	0.328	0.325	0.366	0.421	0.415
Autoformer	∼10 M	0.255	0.339	0.281	0.340	0.339	0.372	0.433	0.432
DLinear-I	130 K	0.246	0.326	0.305	0.371	0.423	0.428	0.624	0.535
**TC-KAN (ours)**	**51 K**	**0.179**	**0.266**	**0.245**	**0.308**	**0.309**	**0.349**	**0.415**	**0.411**
Weather (21 features)									
iTransformer	∼5 M	0.174	0.214	0.221	0.254	0.278	0.296	0.358	0.349
PatchTST	∼10 M	0.177	0.218	0.225	0.259	0.278	0.297	0.354	0.348
TimesNet	∼3 M	0.172	0.220	0.227	0.267	0.286	0.306	0.374	0.353
DLinear-I	130 K	0.196	0.255	0.237	0.296	0.283	0.335	0.345	0.381
**TC-KAN (ours)**	**51 K**	**0.172**	**0.215**	**0.219**	**0.252**	**0.275**	**0.293**	**0.351**	**0.345**

**Table 5:** Ablation study on ETTh1 (*L* = 96 → *H* = 96). Each row removes one component from the full TC-KAN. ΔMSE indicates the percentage increase in MSE relative to the full model.

Configuration	Params	MSE	MAE	ΔMSE
Full TC-KAN	51,465	**0.379**	0.392	—
− Time Conditioning	43,969	0.387	0.392	+2.2%
− KAN (→ FFN)	85,423	0.388	0.392	+2.4%
− CI (→ Shared)	52,239	0.410	0.417	+8.1%
− DepthwiseConv	42,121	0.387	0.392	+2.0%
− RevIN	51,451	0.382	0.398	+0.8%

**Table 6:** Fusion experiment: TC-KAN combined with de-stationary attention under the CI framework. Δ maps the relative MSE change resulting from appending DS-Attention. Bold indicates the lower (better) MSE value in each row.

Dataset	Prediction Horizon (H)	TC-KAN (MSE)	TC-KAN+DS-Attn (MSE)	Δ Change
ETTh2	96	0.290	**0.285**	−1.7%
ETTh2	336	0.427	**0.419**	−1.8%
ETTm1	96	**0.342**	0.339	−0.8%
ETTm1	336	**0.415**	0.408	−1.6%

**Table 7:** Ljung–Box test *p*-values for prediction residuals (*H* = 96). Values >0.05 (bold) indicate the residual sequence is statistically indistinguishable from white noise.

Model	ETTh1	ETTh2	ETTm1	ETTm2	Weather
DLinear-I	0.012	0.003	0.021	0.008	0.034
**TC-KAN (ours)**	**0.158**	**0.074**	**0.121**	**0.095**	**0.142**

## Data Availability

The code and data supporting the findings of this study are available from the corresponding author upon reasonable request.

## Abbreviations

The following abbreviations are used in this manuscript:

LTSF	Long-Term Time Series Forecasting
KAN	Kolmogorov–Arnold Networks
TC-KAN	Time-Conditioned Kolmogorov–Arnold Networks
CI	Channel Independent
RevIN	Reversible Instance Normalization
MSE	Mean Squared Error
MAE	Mean Absolute Error
ETT	Electricity Transformer Temperature
MLP	Multi-Layer Perceptron
FFN	Feed-Forward Network
